# Awareness, Perceptions, and Opinions of Artificial Intelligence Among Undergraduate Medical Students at Umm Al-Qura University, Saudi Arabia, in 2025: A Cross-Sectional Study

**DOI:** 10.7759/cureus.89741

**Published:** 2025-08-10

**Authors:** Bayan Almehmadi, Tahani Mohammadali Bakhsh, Ibrahim Noor Elahi, Nahla Hariri

**Affiliations:** 1 Preventive Medicine, Ministry of Health, Jeddah, SAU; 2 Medicine, Ministry of Health, Jeddah, SAU; 3 Community Medicine and Healthcare of Pilgrims, Umm Al-Qura University, Makkah, SAU

**Keywords:** artificial intelligence, awareness, chatgpt, healthcare technology, medical education, perceptions

## Abstract

Background and objective: The integration of artificial intelligence (AI) education into medical curricula is emphasized by the World Medical Association as AI becomes more prevalent in healthcare, including applications such as image analysis and robotic surgery. This study aimed to assess the awareness, perceptions, and opinions of undergraduate medical students regarding AI and its application in healthcare at Umm Al-Qura University, Makkah, Saudi Arabia.

Materials and methods: A descriptive cross-sectional study was done in March 2025 at Umm Al-Qura University with 170 undergraduate medical students. A stratified random selection method was used to ensure that each year group was represented correctly. Based on earlier studies, a verified, self-administered online questionnaire was used to collect the data. The tool examined demographic information, knowledge, use, attitudes, and impressions of AI. SPSS Statistics version 20 (IBM Corp., Released 2011. IBM SPSS Statistics for Windows, Version 20.0. Armonk, NY: IBM Corp.) was used to do statistical tests such as chi-square tests, independent t-tests, and one-way ANOVA.

Results: Of the 170 students, 70.0% were female and 91.8% were Saudi nationals. A large majority were aware of AI (95.9%) and ChatGPT (97.1%; OpenAI, San Francisco, CA, USA), with 94.1% having used ChatGPT. Most students (61.2%) viewed AI as a supportive tool for healthcare professionals rather than a replacement. Positive perceptions of AI were associated with higher levels of study (p = 0.006) and AI awareness (p = 0.008), but not with gender, age, or nationality. While many students recognized AI’s benefits in diagnosis and decision-making, concerns were expressed regarding dehumanization, job displacement, and ethical risks such as privacy and misinformation. Notably, 55.3% of students reported no formal education on AI, underscoring the need for structured curricular integration.

Conclusions: The study found that Saudi medicine students believed AI was generally beneficial, but they were also cautious. To prepare students for future clinical settings that AI will enhance, it is imperative that formal AI training be included in undergraduate medical education.

## Introduction

Global healthcare is receiving a spotlight because of the use of cutting-edge technology, such as artificial intelligence (AI), to provide high-quality healthcare [1-3]. Though AI performs similar tasks to humans, it cannot perform those duties in the same way; current research examines expanding the capabilities of AI to encompass human capabilities further [4]. AI refers to the study and development of computer systems that can carry out activities that generally require human intelligence, such as speech recognition, visual information perception, taking action, and interpreting languages. In the realms of data, information technology, and science, AI is causing a revolution with its advancements in work efficiency [5].

AI and associated technologies are more pervasive in several facets of human existence and are beginning to impact the healthcare sector as well [6]. AI tools have been designed to gather and evaluate healthcare data, including records about patients, behaviors, the environment, and drugs, using information from medical research [7]. Using convolutional neural networks has made the detection of diabetic retinopathy, diagnosis of skin lesions, identification of lymph node metastasis, and spotting of problems in radiographs more efficient [8-11].

According to Kreps and Neuhauser (2013), AI has the potential to improve patients' quality of life by assisting them in comprehending their symptoms and influencing their actions while seeking medical attention [12]. AI assistants have suggested cancer therapies that are as effective as or more effective than those recommended by human experts [13]. A skilled AI assistant can quickly sort and analyze enormous amounts of data, make inferences, and greatly expand its use in medical research. With advancements such as AI-driven surgeries, virtual helping assistants, and sophisticated image analysis, artificial intelligence is poised to have a profound impact on healthcare. Essentially, AI can assist healthcare professionals and patients in several key areas, including predicting treatment outcomes, managing complications, diagnosing illnesses, assessing risks, and providing patient support [14].

AI is a quickly evolving field that will transform medical research and become an essential part of medicine in the future. Despite predictions that AI will play a significant role in medical education in the next 10 years [15], little is known about the factors that influence medical students' intentions to adopt AI technologies [16] or their attitudes towards the use of AI in clinical practice [17]. Consequently, it is essential to find out how medical students feel about AI and what they intend to accomplish.

Many countries are increasing their use of AI in healthcare systems to enhance the delivery of health services [18]. AI, machine learning, neural networks, and deep learning [2] are expected to play a role in diagnosing, making predictions, and therapy in the medical community [19,20]. The increase in medical use of AI brings about additional ethical considerations [21]. Because of the pandemic, AI now helps medical education by providing students with opportunities to practice complex medical actions on virtual people, which reduces the risk of harming patients [22].

With AI expected to revolutionize medical services, it is essential to assist healthcare professionals and medical students in adapting, which involves examining their views [23]. Several studies looked into the level of awareness and belief about AI among medical and healthcare professionals or medical students in various regions [24]. Furthermore, study findings exhibit inconsistency about the disparities in awareness, beliefs, and views related to technology use. Conversely, medical students are encountering purported obstacles posed by the fast advancement of AI and express apprehensions over potential replacement by future AI technologies [25]. This problem may confound medical students' perceptions of AI in medical care. Research indicates that AI may diminish training possibilities for the development of "clinical judgment" and "practical skills" among student doctors [26].

Recent developments in AI in the healthcare sector demonstrate that physicians and medical students need thorough education in AI. Thus, enhancing understanding of AI among prospective healthcare professionals is essential to inform their career decisions. This subject has garnered considerable attention; nonetheless, it remains comparatively underexplored in the Middle East and Arab nations. There is a paucity of evidence concerning Arab medical students' understanding, attitudes, and opinions regarding artificial intelligence and deep learning in medicine and radiology. Additionally, it is essential to examine their perspectives on the incorporation of AI in medical school curricula and analyze any disparities between Arab and international medical students. Therefore, this study aims to investigate artificial intelligence awareness, perceptions, and opinions among undergraduate medical students at Umm Al-Qura University, Makkah, Saudi Arabia.

## Materials and methods

A cross-sectional approach was conducted between January 1 and April 26, 2025, to study the group of undergraduate medical students at Umm Al-Qura University. The study was designed to examine students' awareness, perceptions, and opinions about the use of AI in healthcare. With approval, the survey methodology was changed based on prior research that had been verified in Riyadh and checked by experts in preventive medicine to ensure that it was relevant to the situation. The English-language self-administered questionnaire had four parts: demographic information, knowledge about AI, attitudes about AI, and thoughts about AI in medical practice. Before the complete implementation, a pilot test with 10% of the sample (n = 17) was done to examine for clarity and internal consistency; these participants were included in the final analysis. A Cronbach's alpha of 0.840 showed that the 18-item Likert scale used to measure perceptions was reliable internally. A total of 170 undergraduate students participated in the survey, ensuring a diverse representation from each year of the MBBS program. With a 5% margin of error, numbers from the university's academic office and a 95% confidence range were used to figure out the sample size for the Raosoft software estimate from the parent article [27]. Class leaders sent out survey invites via WhatsApp and email to ensure all students were represented throughout the school year. Participants filled out the survey online via SurveyMonkey (Momentive Inc., San Mateo, CA, USA). For clarity and openness, the results section shows percentage values together with the sample sizes (n) that go with them. The inter- and non-medical students were excluded from the study.

After getting informed consent from all participants, data were gathered without their names being known. The project was approved by the Biomedical Research Ethics Committee (approval number: HAPO-02-K-012-2024-12-2417), and steps to keep participant data private were taken as defined by the IRB. SPSS Statistics version 20 (IBM Corp., Released 2011. IBM SPSS Statistics for Windows, Version 20.0. Armonk, NY: IBM Corp.) was used to manage and analyze the data. A summary of the answers was made using descriptive data like means, rates, and standard deviations. We used Pearson's chi-squared test to examine the relationships between demographic factors and AI awareness. The perception scores of different classes were compared using independent samples t-tests and one-way ANOVA. We conducted a Kolmogorov-Smirnov test to see if the data were normal. It was decided that p < 0.05 was the cutoff for statistical significance.

## Results

The overall research indicated that people of various ages, genders, nationalities, and academic years were equally aware of AI, with no statistically significant variances in awareness based on these factors. However, students' opinions of AI varied greatly depending on their degree of study and how familiar they were with it. The evidence suggests that increased familiarity with AI and a more in-depth study of it led to more positive opinions.

Demographic characteristics

Table 1 shows demographic data of the 170 participating students, revealing a predominantly female population, with 70.0% identifying as female and 30.0% as male. The majority of the respondents (72.4%) are undergraduates. They must be in this age group because medical school admissions typically occur between the ages of 17 and 18. Nationality-wise, an overwhelming 91.8% of the students are Saudi nationals, while only 8.2% are from other countries, highlighting a strong local representation in the study. Regarding academic level, the students are fairly distributed across different years of study. The second year had the highest representation at 28.2%, followed by the third year (18.8%), and both the first and sixth years contributed equally, each at 18.2%. The fourth and fifth years had lower representation at 10.0% and 6.5%, respectively. This distribution reflects a balanced participation from both junior and senior academic levels, adding diversity to the sample in terms of educational experience.

**Table 1:** Demographic characteristics of the students (n = 170)

Variables	Category	Frequency (n)	Percentage (%)
Gender	Male	51	30.0%
	Female	119	70.0%
Age (years)	18–22	123	72.4%
	23–25	34	20.0%
	26–30	13	7.6%
Nationality	Saudi	156	91.8%
	Non-Saudi	14	8.2%
Level of study	First year	31	18.2%
	Second year	48	28.2%
	Third year	32	18.8%
	Fourth year	17	10.0%
	Fifth year	11	6.5%
	Sixth year	31	18.2%

Students’ awareness of AI

Table 2 illustrates the impressive level of awareness and active engagement among students regarding AI and ChatGPT. A vast majority of students (95.9%) were aware of AI, indicating that the concept has been widely recognized in academic circles. Even more striking is the awareness of ChatGPT, with 97.1% of respondents acknowledging familiarity with the term, showcasing its growing presence in the digital learning environment. In terms of practical usage, 94.1% of the students have already used ChatGPT, reflecting a strong inclination towards integrating AI tools into their academic or personal routines. This high usage rate suggests that ChatGPT is not only a well-known platform but also a valuable resource embraced by students for learning, research, and productivity. The minimal percentage of students who are unaware of or have not used AI or ChatGPT further illustrates the profound effect of these technologies on the student community.

**Table 2:** Awareness and usage of AI and ChatGPT among students (n = 170) AI: artificial intelligence

Questions	Response	Frequency (n)	Percentage (%)
Are you aware of AI?	Yes	163	95.9%
	No	7	4.1%
Are you aware of the term ChatGPT?	Yes	165	97.1%
	No	5	2.9%
Have you used ChatGPT?	Yes	160	94.1%
	No	10	5.9%

Student opinions regarding AI

Table 3 provides valuable insight into students’ perspectives on the role of AI in healthcare, as well as their educational background related to AI. When asked whether AI would replace physicians in the healthcare system, the majority of students (61.2%) viewed AI as a supportive tool rather than a replacement, indicating a balanced understanding of AI's potential as an aid to medical professionals. Meanwhile, 21.2% of respondents believed that AI could replace physicians, and 13.5% disagreed with this notion. A small proportion (4.1%) expressed uncertainty.

**Table 3:** Students’ opinions and educational background regarding AI (n = 170) AI: artificial intelligence

Questions	Response options	Frequency (n)	Percentage (%)
Do you think that AI will replace the physician in the healthcare system?	Agree	36	21.2%
	Disagree	23	13.5%
	I don’t know	7	4.1%
	It is a tool that helps healthcare professionals	104	61.2%
What is your opinion if AI becomes widespread in Saudi Arabia?	Healthcare professionals will be better with the widespread use of AI	75	44.1%
	Risk of job loss due to AI/robots	45	26.5%
	I don't know	13	7.6%
	Specialization choices will depend on AI use in that field	37	21.8%
Have you received any formal education about AI?	Yes	39	22.9%
	No	94	55.3%
	Received training over the internet	25	14.7%
	Received training through seminars and presentations	12	7.1%

Regarding the potential impact of AI’s widespread adoption in Saudi Arabia, 44.1% of students believed it would enhance healthcare professionals' performance. However, concerns were also evident, with 26.5% expressing fear of job loss due to automation and robotics. Additionally, 21.8% noted that the integration of AI in specific fields may influence future specialization choices, while 7.6% remained unsure. In terms of formal education, more than half of the students (55.3%) reported having no formal education on AI. However, a portion had engaged in self-directed learning, with 22.9% having received formal instruction, 14.7% receiving training via the internet, and 7.1% attending seminars or presentations. These results suggest that while students are aware of and interested in AI, there remains a gap in structured educational opportunities, highlighting the need for more formal AI integration into academic curricula.

Perceptions of the students about AI

Table 4 shows a thorough overview of what students think about the benefits, risks, and ethics of AI being used in healthcare. A large number of participants express that AI has a positive influence on medical practices. The majority of the participants (57.1%) agreed that AI makes it easier to diagnose and manage diseases, and an even larger number (71.2%) felt that AI supports professionals in making better decisions when treating people. More than 80% of students agreed or wholeheartedly agreed that AI makes it easier for patients to get and understand information. At the same time, responses pointed out a cautious recognition that AI can have its limitations and risks. About 55% were worried that AI may weaken the standing of the profession or change its caring nature, and 47.1% said it could lead to less trust between patients and medical professionals. Similarly, more than half (54.7%) acknowledged potential privacy violations, misinformation risks, and bias associated with AI technologies.

**Table 4:** Perceptions of the students about AI (n = 170) AI: artificial intelligence

Variables	Strongly agree n (%)	Agree n (%)	Neutral n (%)	Disagree n (%)	Strongly disagree n (%)
AI devalues the medical profession	6 (3.5%)	47 (27.6%)	51 (30.0%)	47 (27.6%)	19 (11.2%)
AI reduces errors in medical practice	22 (12.9%)	75 (44.1%)	51 (30.0%)	17 (10.0%)	5 (2.9%)
AI facilitates healthcare professionals’ access to information	48 (28.2%)	90 (52.9%)	24 (14.1%)	6 (3.5%)	2 (1.2%)
AI enables healthcare professionals to make more accurate decisions	25 (14.7%)	84 (49.4%)	48 (28.2%)	10 (5.9%)	3 (1.8%)
AI increases patients’ confidence in medicine	14 (8.2%)	54 (31.8%)	68 (40.0%)	30 (17.6%)	4 (2.4%)
AI facilitates patient education	26 (15.3%)	92 (54.1%)	42 (24.7%)	10 (5.9%)	0 (0%)
AI negatively affects the relationship between healthcare professionals and the patient	13 (7.6%)	47 (27.6%)	55 (32.4%)	44 (25.9%)	11 (6.5%)
AI damages the trust that is the basis of the patient-healthcare professional relationship	10 (5.9%)	56 (32.9%)	56 (32.9%)	39 (22.9%)	9 (5.3%)
AI reduces the humanistic aspect of the medical profession	19 (11.2%)	62 (36.5%)	49 (28.8%)	32 (18.8%)	8 (4.7%)
AI violation of professional confidentiality may occur more	23 (13.5%)	70 (41.2%)	48 (28.2%)	25 (14.7%)	4 (2.4%)
AI allows the patient to increase his control over his health	18 (10.6%)	74 (43.5%)	48 (28.2%)	27 (15.9%)	3 (1.8%)
AI can improve healthcare by streamlining diagnoses and improving clinical outcomes	18 (10.6%)	97 (57.1%)	44 (25.9%)	7 (4.1%)	4 (2.4%)
The algorithm can assess large amounts of data about a person's medical history to determine the best treatment plan	34 (20.0%)	87 (51.2%)	41 (24.1%)	7 (4.1%)	1 (0.6%)
AI methods (ML/DL) are widely used in the prediction and diagnosis of diseases	19 (11.2%)	73 (42.9%)	71 (41.8%)	4 (2.4%)	3 (1.8%)
An artificial neural network predicts chronic diseases (e.g., diabetes, cancer, heart disease)	10 (5.9%)	65 (38.2%)	72 (42.4%)	20 (11.8%)	3 (1.8%)
Major dangers of AI include misinformation (e.g., deep fakes)	42 (24.7%)	69 (40.6%)	44 (25.9%)	13 (7.6%)	2 (1.2%)
Drawbacks of AI include privacy concerns, bias, and discrimination	23 (13.5%)	76 (44.7%)	51 (30.0%)	18 (10.6%)	2 (1.2%)
AI facilitates patients’ access to the service	28 (16.5%)	93 (54.7%)	38 (22.4%)	10 (5.9%)	1 (0.6%)

Interestingly, a large portion of students recognized AI’s analytical strengths, with 71.2% agreeing that algorithms can evaluate vast medical histories to support treatment planning. The use of machine learning and deep learning in disease prediction was also widely acknowledged (54.1% agreement). While concerns about job displacement and loss of professional autonomy were evident, the prevailing sentiment supports the integration of AI as a tool to complement rather than replace human healthcare providers. These findings suggest that students are both optimistic and discerning in their views on AI, appreciating its ability to enhance healthcare delivery while remaining alert to its ethical and professional challenges.

Reliability analysis

The reliability of the questionnaire, which consisted of 18 Likert scale items, was evaluated using Cronbach’s alpha (Table 5). The Cronbach’s Alpha based on standardized items was 0.841, further confirming the reliability of the scale. These results demonstrate that the Likert scale items consistently measure the underlying construct and are suitable for further analysis.

**Table 5:** Reliability statistics of the questionnaire

Reliability measure	Value	Number of items
Cronbach’s alpha	0.840	18
Cronbach’s alpha based on standardized items	0.841	18

Chi-square test of independence

The chi-square analysis in Table 6 shows that there is no meaningful link between students' awareness of AI and factors like gender (p = 0.077), age (p = 0.060), nationality (p = 0.418), or level of study (p = 0.361). Although awareness was generally high across all groups, with male, non-Saudi, and sixth-year students reporting 100% awareness, these differences were not significant. This evidence suggests that AI awareness is consistently high among students, regardless of demographic background.

**Table 6:** Awareness of AI by demographic characteristics (n=170) The table displays the number of undergraduate medical students (n = 170) who are aware of AI, categorized by their demographic factors, with responses indicated as "yes" or "no." Values are shown as a proportion of the total. We also conducted the chi-square (χ²) test to determine if there was a link between each demographic factor and knowledge of AI. A p-value of less than 0.05 means that the result is statistically significant. AI: artificial intelligence

Demographic variable	Category	Awareness of AI				
		Yes n (%)	No n (%)	Total n (%)	Chi-square (χ²)	p-value
Gender	Male	51 (100%)	0 (0%)	51 (100%)	3.129	0.077
	Female	112 (94.1%)	7 (5.9%)	119 (100%)		
Age (years)	18–22	118 (95.9%)	5 (4.1%)	123 (100%)	5.641	0.060
	23–25	34 (100%)	0 (0%)	34 (100%)		
	26–30	11 (84.6%)	2 (15.4%)	13 (100%)		
Nationality	Saudi	149 (95.5%)	7 (4.5%)	156 (100%)	0.655	0.418
	Non-Saudi	14 (100%)	0 (0%)	14 (100%)		
Level of study	First year	30 (96.8%)	1 (3.2%)	31 (100%)	5.472	0.361
	Second year	47 (97.9%)	1 (2.1%)	48 (100%)		
	Third year	30 (93.8%)	2 (6.2%)	32 (100%)		
	Fourth year	15 (88.2%)	2 (11.8%)	17 (100%)		
	Fifth year	10 (90.9%)	1 (9.1%)	11 (100%)		
	Sixth year	31 (100%)	0 (0%)	31 (100%)		

Independent sample t-test and one-way ANOVA

The analysis in Table 7 using independent t-tests and a one-way ANOVA showed that there were no significant differences in average positive perception scores towards AI based on gender, age, or nationality (p > 0.05). However, the level of study showed a statistically significant difference (p = 0.006), indicating that perceptions vary across academic years, with fourth- and sixth-year students having higher positive perceptions. Additionally, awareness of AI was significantly associated with positive perceptions (p = 0.008), suggesting that those who were aware of it tended to view it more favorably.

**Table 7:** Association between mean positive perception score of AI and participant characteristics (n = 170) ^a ^Independent sample t-test, ^b ^one-way ANOVA AI: artificial intelligence, ANOVA: analysis of variance

Variables	Categories	Mean	Std. dev	F-value	t-value	p-value
Gender	Male	2.09	0.64	-	0.153	0.878^a^
	Female	2.07	0.56			
Age (years)	18–22	2.02	0.55	1.769	-	0.174^b^
	23–25	2.23	0.58			
	26–30	2.14	0.86			
Level of study	First year	2.01	0.57	3.393	-	0.006^b^
	Second year	1.85	0.51			
	Third year	2.12	0.55			
	Fourth year	2.3	0.58			
	Fifth year	2.08	0.48			
	Sixth year	2.32	0.66			
Nationality	Saudi	2.09	0.64	-	-0.447	0.655^a^
	Non-Saudi	2.07	0.56			
Awareness of AI	Yes	2.09	0.64	-	-1.894	0.008^a^
	No	2.07	0.56			

## Discussion

Despite significant research on the application of AI in medical education, the perspectives of medical professionals and their challenges regarding its incorporation into everyday practice remain relatively underexplored. This research examines the awareness, perceptions, and opinions of undergraduate medical students regarding the use of AI. Limited literature exists both nationally and internationally regarding undergraduates' awareness, perceptions, and opinions of AI in healthcare; however, the majority of the available studies focus on practicing healthcare professionals and medical students [28-30]. This study offers necessary information regarding the readiness of future physicians to adapt to AI-integrated healthcare and may provide a baseline for subsequent national and regional research.

Our research revealed that a large percentage of students (95.9%) are aware of AI, signifying that the concept has gained extensive recognition within academic spheres. 97.1% of respondents recognized the term ChatGPT, indicating its increasing prominence in the digital learning landscape. Practically, 94.1% of students have used "ChatGPT," indicating a significant propensity for incorporating AI technology into their academic or personal activities. In another study, approximately 70% of medical students and 81.8% of physicians were aware that AI could soon assist doctors. The majority of those surveyed believed that AI would provide support rather than surpass them [31].

This study reveals that 17.8% of students believe AI has the potential to replace nurses, chemists, and physicians in the medical field. Nearly 50% of the participants agreed with the findings that AI will lead to fewer employment options for healthcare workers [32]. According to a different survey, 79.4% of American students said they would like to use AI technology, and 91.5% of them believed that learning about it in medical school would help them in the future [33].

According to Al Hadithy et al. (2023), students thought that AI technology could improve health by personalizing care based on patient data. Additionally, 54.7% of the participants expressed concerns about privacy issues and the risk of false information, which aligns with global findings from the WHO and the EU’s Trustworthy AI Guidelines on the ethical use of AI in healthcare systems [34].

Consistent with earlier results, over half of the students (55.3%) reported no formal training in AI. However, Paranjape et al. pointed out that medical schools have failed to teach students about the intricacies of EHR systems' backends, including data quality, the impact of computer use on patient interactions, and the complexities of the doctor-patient relationship [35]. When used with sufficient training and comprehension, AI will liberate physicians' time and optimize their work hours, enabling them to engage with and care for patients during their spare time [32]. This inadequacy renders medical students ill-equipped to use AI technology in their future practice. Our research highlights the necessity for curricular change, especially within Saudi medical schools.

Our research revealed that fourth- and sixth-year students exhibited significantly more positive attitudes towards AI (p = 0.006), supporting the results found by Chai et al. (2021), which indicated that senior medical students had higher levels of AI-related self-efficacy and a greater willingness to use the technology. This study reinforced the findings by showing that intention-related attitudinal components may be influenced by perceived self-efficacy and highlighting the connection between attitude towards behavior and perceived behavioral control. Since basic knowledge and subjective norms were identified as predictors of self-efficacy, the study concluded that verbal communications and vicarious experiences are two critical components that contribute to self-efficacy [36].

The perception scale's internal consistency, evidenced by a Cronbach’s alpha of 0.840, confirms the reliability of these results. The study revealed no significant correlation between demographic characteristics and awareness of AI, indicating that understanding of AI varies significantly across gender, age, and nationality among medical undergraduates. This collective understanding, irrespective of background, may indicate the widespread incorporation of digital technologies such as ChatGPT into student life and academic practices. Conversely, Syed et al. (2023) indicated that the average favorable opinion of AI (29.8 ± 9.63) was strongly affected by age, academic year, and nationality, while showing no significant link with AI awareness. Their findings indicate that senior students and older individuals often possess more positive attitudes, possibly attributable to greater exposure through official AI-related activities, including courses, seminars, and conferences [27]. These variations may indicate variations in curricular exposure or technology integration among schools.

Despite considerable knowledge among research participants, the notable correlation between years of study and perception evaluations (p = 0.006) confirms that educational maturity may augment receptiveness to AI. Consequently, although knowledge may be evenly dispersed, the cultivation of favorable opinions seems more directly associated with educational advancement and formal interactions with AI content. These results can help plan targeted AI training in undergraduate medical education, particularly in the early years, to tackle differences in perceptions and build lasting skills in digital health technologies.

Enhancing the self-efficacy of medical students is essential for increasing their enthusiasm for medical AI studies. By enhancing their comprehension of medical AI and providing a supportive subjective norm regarding it, their confidence in learning AI for medical uses may improve. To sum up, the rise of AI has profoundly impacted both medical science and clinical practice. Researchers [36-38] argue that medical education needs to incorporate AI-enhanced approaches and undergo vital changes. Medical students need to prepare themselves with knowledge of AI technologies for clinical use.

Motivating students to understand AI is crucial, primarily since they will be functioning in a society that is enhanced by AI in the future [39]. This study suggests that mentors and medical institutions can foster positive subjective norms by exposing students to medical AI knowledge. Medical students may therefore have a strong desire to study AI for medical practice after seeing the importance of medical AI in their careers and feeling more confident about learning about it.

When interpreting the findings of this investigation, it is crucial to take into account several limitations. The study was conducted among undergraduate medical students at one institute; consequently, the findings may not apply to students from different medical institutions or fields, whether in Saudi Arabia or elsewhere. Variations in curricular exposure, institutional culture, and access to AI resources among institutions with a small sample size may limit the generalizability of these findings. The omission of these health professionals hindered a detailed comparison of the differences in AI awareness and perception across healthcare disciplines. Additionally, the accuracy and honesty with which participants report their answers on a self-completed online questionnaire can lead to response bias.

Data is gathered during cross-sectional analysis at a given time, which prevents research from showing cause-and-effect relationships or tracking changes in opinions. To carry out the study, respondents were tapped through institutional networks and random selection, which makes the results reliable but is still vulnerable to choice bias for those who use digital tools more frequently. The study might have some shortcomings, but it sheds light on students’ perspectives and thoughts about AI, and it also stresses the need to address some areas of educational deficiency. These results can serve as a key guideline for educators and officials seeking to enhance students’ AI knowledge in medical school programs across the Kingdom.

## Conclusions

Saudi medical undergraduates demonstrate a strong understanding of AI, holding mixed opinions that reflect both hope for its medical benefits and concerns about its ethical and professional implications. Such findings are supported by worldwide research and highlight the need for properly teaching AI in medical programs to prepare future doctors for collaborating with AI in healthcare.

## Appendices

**Table 8:** Questionnaire AI: artificial intelligence

A. Demographic characteristics					
1. Gender	☐ A. Male	☐ B. Female			
2. Age in years	☐ A. 18–22	☐ B. 23–25	☐ C. 26–30		
3. Nationality	☐ A. Saudi	☐ B. Non-Saudi			
4. Level of study	☐ A. First year	☐ C. Third year	☐ F. Fifth year		
	☐ B. Second year	☐ D. Fourth year	☐ i. Sixth year		
B. Awareness of students about AI					
	Yes	No			
1. Are you aware of AI?	☐	☐			
2. Are you aware of the term ChatGPT?	☐	☐			
3. Did you use ChatGPT?	☐	☐			
C. Opinions of students about AI					
1. Do you think that AI will replace the physician?	☐ A. Agree	☐ B. Disagree	☐ C. It is a tool that helps professionals	☐ D. I don't know	
2. What is your opinion on whether AI is widespread in Saudi Arabia?	☐ A. Risk of job loss due to robots	☐ B. Better for professionals	☐ C. Affects specialization choice	☐ D. I don't know	
3. Have you received formal education about AI?	☐ a. Yes	☐ b. No	☐ c. Internet training	☐ d. Seminars/presentations	
D. Perceptions of students about AI					
	Strongly Disagree	Disagree	Neutral	Agree	Strongly Agree
1. AI devalues the medical profession?	☐	☐	☐	☐	☐
2. AI reduces errors in medical practice?	☐	☐	☐	☐	☐
3. AI facilitates patients’ access to the service?	☐	☐	☐	☐	☐
4. AI facilitates professionals’ access to information?	☐	☐	☐	☐	☐
5. AI enables accurate decisions?	☐	☐	☐	☐	☐
6. AI increases patient confidence?	☐	☐	☐	☐	☐
7. AI facilitates patient education?	☐	☐	☐	☐	☐
8. AI negatively affects professional-patient relationship?	☐	☐	☐	☐	☐
9. AI damages trust in the professional relationship?	☐	☐	☐	☐	☐
10. AI reduces the humanistic aspect?	☐	☐	☐	☐	☐
11. AI may cause confidentiality violations?	☐	☐	☐	☐	☐
12. AI allows patient to control own health?	☐	☐	☐	☐	☐
13. AI improves diagnosis and clinical outcomes?	☐	☐	☐	☐	☐
14. Algorithms assess medical history for treatment?	☐	☐	☐	☐	☐
15. AI (ML/DL) used in disease prediction?	☐	☐	☐	☐	☐
16. ANN predicts chronic diseases?	☐	☐	☐	☐	☐
17. AI dangers include misinformation and deepfakes?	☐	☐	☐	☐	☐
18. AI drawbacks include privacy and bias.	☐	☐	☐	☐	☐
